# detectIR: A Novel Program for Detecting Perfect and Imperfect Inverted Repeats Using Complex Numbers and Vector Calculation

**DOI:** 10.1371/journal.pone.0113349

**Published:** 2014-11-19

**Authors:** Congting Ye, Guoli Ji, Lei Li, Chun Liang

**Affiliations:** 1 Department of Automation, Xiamen University, Xiamen, Fujian 361005, China; 2 Department of Biology, Miami University, Oxford, Ohio 45056, United States of America; 3 Innovation Center for Cell Biology, Xiamen University, Xiamen, Fujian 361005, China; 4 State Key Laboratory for Biology of Plant Diseases and Insect Pests, Institute of Plant Protection, Chinese Academy of Agricultural Sciences, Beijing 100193, China; Universita’ di Padova, Italy

## Abstract

Inverted repeats are present in abundance in both prokaryotic and eukaryotic genomes and can form DNA secondary structures – hairpins and cruciforms that are involved in many important biological processes. Bioinformatics tools for efficient and accurate detection of inverted repeats are desirable, because existing tools are often less accurate and time consuming, sometimes incapable of dealing with genome-scale input data. Here, we present a MATLAB-based program called *detectIR* for the perfect and imperfect inverted repeat detection that utilizes complex numbers and vector calculation and allows genome-scale data inputs. A novel algorithm is adopted in *detectIR* to convert the conventional sequence string comparison in inverted repeat detection into vector calculation of complex numbers, allowing non-complementary pairs (mismatches) in the pairing stem and a non-palindromic spacer (loop or gaps) in the middle of inverted repeats. Compared with existing popular tools, our program performs with significantly higher accuracy and efficiency. Using genome sequence data from HIV-1, *Arabidopsis thaliana*, *Homo sapiens* and *Zea mays* for comparison, *detectIR* can find lots of inverted repeats missed by existing tools whose outputs often contain many invalid cases. *detectIR* is open source and its source code is freely available at: https://sourceforge.net/projects/detectir.

## Introduction

An inverted repeat is a nucleotide sequence fragment that can form self-complementary pairing between its two halves. The perfect inverted repeats are also known as palindrome where one of these two halves is exactly the reverse complement of the other; in contrast, imperfect inverted repeats contain nucleotide pairs that are not reversely complementary (*i.e.*, mismatched), often with a non-palindromic spacer (loop or gaps) in the middle [1] [2] [3]. Abundant inverted repeats are present in both prokaryotic and eukaryotic genomes with nonrandom distributions, and they are involved in many biological processes including DNA replication [4], DNA transition [5] and DNA methylation [6]. In yeast, long inverted repeats were demonstrated to be mitotic recombination hotspots, and quasipalindromes (imperfect inverted repeats) underwent deletion more frequently [7]. In mouse embryonic stem cells, inverted repeats get involved in the generation of unstable chromosomal rearrangements [8]. Inverted repeats of > = 6 complementary nucleotides, either perfect or imperfect, can form secondary structures – cruciforms in double stranded DNA [9]. Some DNA-binding proteins have their two binding sites arranged as in an inverted repeat [3] [4] [10]. Using atomic force microscopy images, the DNA-binding protein PARP-1 was shown to bind the cruciform structure generated by a 106-*nt* inverted repeat within an *E. coli* plasmid [5]. PARP-1 was found to participate in chromatin structure coordination and gene expression regulation [11], and it did show a binding preference to cruciform structures than loops or linear DNAs [9]. In humans, a 14-*nt* imperfect inverted repeat sequence located in distal promoter region of human HFE gene can form a cruciform structure that binds PARP-1 protein to repress HFE transcription, and increased ion level can trigger PARP-1 breakdown to release such transcriptional repression [12]. Interestingly, using 2D electrophoretic analysis of DNA replication intermediates, single-stranded hairpins formed by imperfect inverted repeats with a central non-palindromic spacer, rather than double-stranded cruciforms, proved to be responsible for replication stalling that induces genome instability [13]. On the other hand, as a part of gene expression production, hairpin is a common and important secondary structural element in RNA transcripts, and its single-stranded template DNA must have a relevant inverted repeat [3], suggesting that inverted repeats in transcribed DNAs might play a potential regulatory role in resultant RNA transcripts. For instance, in yeast and mammalian pre-mRNAs, inverted repeats located in introns were shown to affect alternative splicing [14] [15] [16]. Moreover, inverted repeats have been utilized for gene silencing in fungi and plants for many years [17] [18] [19]. They also delineate transposon element boundaries. For instance, miniature inverted repeat transposable elements (MITEs) are characterized by their terminal inverted repeats [20]. Genes away from MITEs show higher expression than those that contain or are close to MITEs [21]. Clearly, the role of inverted repeats in gene expression regulation is worthy of further wet-lab experimental investigation and validation. The identification and characterization of inverted repeats at genome-wide scale will offer an important glimpse and survey that will facilitate our understanding of inverted repeats and their biological functions.

Lu et al. examined the distribution of perfect inverted repeats (palindromes) in human genome [22]. They found that palindromes show higher abundance in introns than exons while upstream regions (*i.e.*, 2,000 bp upstream from translational start site) also contain rich palindromes that can serve as binding sites for transcription factors. Interestingly, they also scanned the human genome for imperfect inverted repeats (*i.e.*, near-perfect palindromes of < = 4 mismatches between two halves, with a short spacer in the middle) and found a similar distribution pattern as perfect inverted repeats [22]. In yeast *Saccharomyces cerevisiae* genome, both palindromes and imperfect inverted repeats (*i.e.*, the pairing stem length >6 *nt* and the spacer length less than 77 *nt*) were significantly richer than randomized genome [3]. In particular, imperfect inverted repeats with short spacers, which have a greater susceptibility to cruciform extrusion than long spacers, were significantly enriched in intergenic regions near 3′ gene ends than near 5′ gene ends [3]. Using yeast relatives *S. paradoxus*, *S. mikatae* and *S. bayanus*, Humphey-Dixon and coworkers studied the conservation of both perfect and imperfect inverted repeats in yeast *S. cerevisiae* genome, and they found that both conserved inverted repeats in promoters and inverted repeats in the promoters of highly expressed genes are most frequently located near the transcriptional start sites, indicating their potential function in transcriptional regulation [23]. As more and more genome sequences are available for different species, it is important for us to conduct genome-wide comparative study to determine the distributions, properties, and conservation of inverted repeats among different species, either distantly or closely related, in order to deepen our understanding of inverted repeats and their biological importance.

To better understand the roles of inverted repeats in genome organization and evolution, developing efficient programs to conduct genome-wide detection of inverted repeats is particularly important. Recently, a MATLAB-based tool *findIR* [24] was created for detecting perfect inverted repeats. In comparison with the existing similar tools that adopt conventional string comparison algorithms, *findIR* deployed a novel algorithm that uses prime number scoring system and turns sequence search into the calculation, search and comparison of numbers. Consequently, *findIR* proved to have obviously higher accuracy in detecting perfect inverted repeats than several popular tools, and it was capable of processing genome-scale inputs. Unfortunately, it is difficult to use prime numbers to represent imperfect inverted repeats that contain non-complementary pairs in the pairing stem (*i.e.*, two halves) and a central non-palindromic spacer. The search and validation strategy of *findIR* is not designed for detecting imperfect inverted repeats. Moreover, *findIR* is limited to detect perfect inverted repeats of length shorter than 1,000 *nt* and the robust vector calculation power of MATLAB has not been utilized at all by *findIR* to enhance the program efficiency.

Here, we developed a novel program *detectIR* that also turns sequence search into numerical calculation and manipulation using complex numbers, which can represent both perfect and imperfect IRs accurately and efficiently. In comparison with *findIR*, the novelty of *detectIR* lies in a novel mapping schema that utilizes complex numbers, a distinctive and effective strategy of search and validation to evaluate candidates of both perfect and imperfect inverted repeats, and the utilization of MATLAB built-in vector calculation power that enables simultaneous detection of inverted repeats of same length to improve the program efficiency significantly.

## Design and Implementation

**Figure 1:**
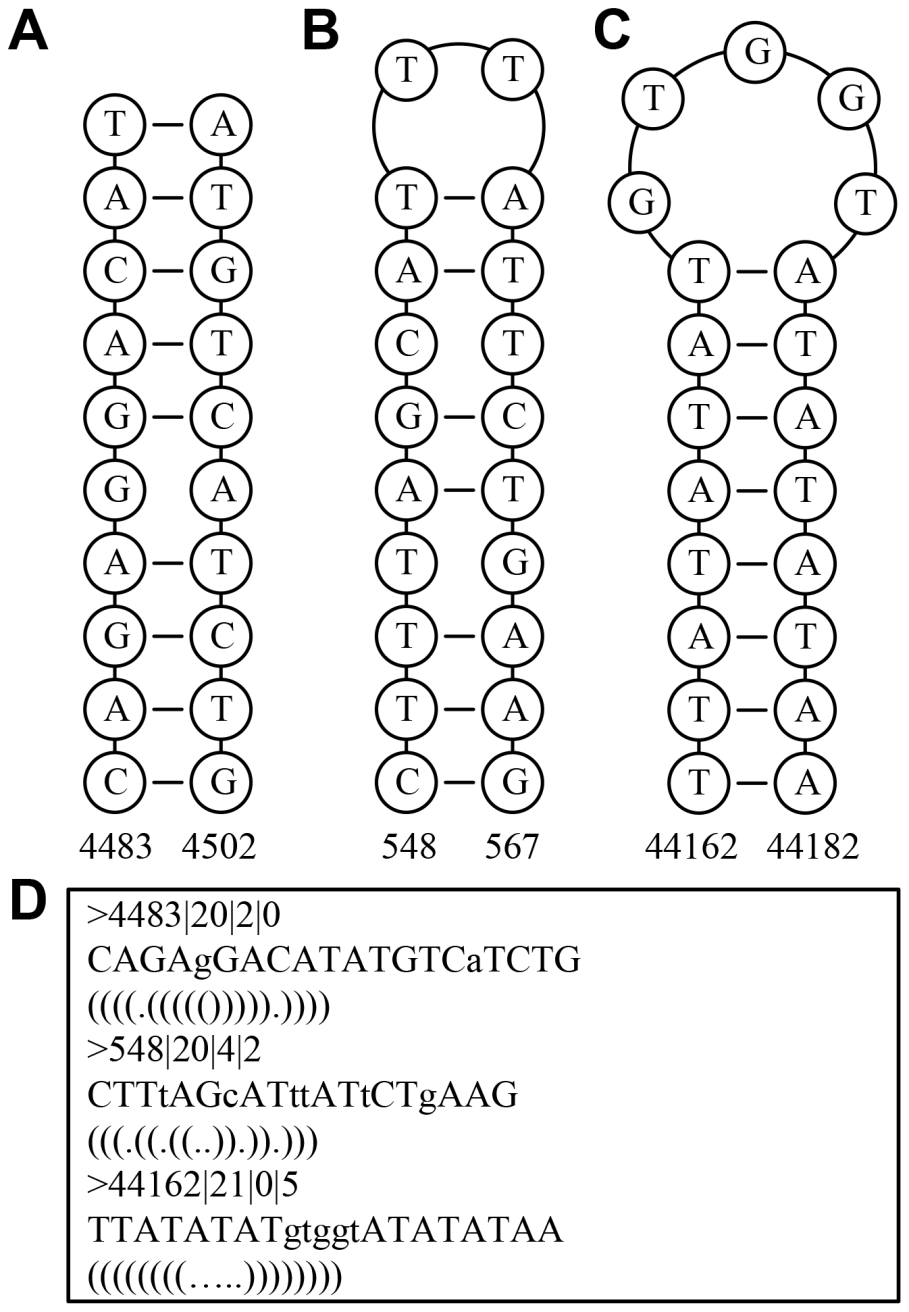
Examples of imperfect inverted repeats detected in the chromosome 1 *of Arabidopsis thaliana* by *detectIR*. Circles highlight the nucleotide bases and numbers represent the start and end genomic coordinates of the imperfect inverted repeats. (A) An imperfect inverted repeat containing only mismatches (un-pairing pair). (B) An imperfect inverted repeat containing both mismatches and a spacer (gaps). (C) An imperfect inverted repeat containing only a spacer (gaps) in the middle. (D) The *detectIR* output for the aforementioned 3 imperfect inverted repeats in the combined format of dot-bracket notation and FASTA. The dots represent the spacer or mismatch nucleotides while brackets indicate the pairing relations. The FASTA description line (*e.g.*, >4483|20|2|0) has the following explanation: >GenomicStartPosition|InvertedRepeatLength|MismatchNumber|GapNumber.

Most existing bioinformatics tools in inverted repeat detection rely on string comparison that is often computational resource demanding and less accurate. Using a novel algorithm that employs prime number scoring system and numerical calculation and search to detect perfect inverted repeats (or palindromes), *findIR* was demonstrated to have much higher accuracy than BioPHP (http://www.biophp.org/minitools/find_palindromes), MATLAB built-in *palindromes* function and EMBOSS *palindrome* tool [25] in detecting perfect inverted repeats. However, both EMBOSS *palindrome* tool and MATLAB built-in *palindromes* function can detect imperfect inverted repeats that contain a central non-palindromic spacer (loop) and/or non-complementary pairs in the pairing stem. In the core algorithm of *findIR*, nucleotide bases are first mapped to a prime number scoring system in which scores of reversely complementary bases can cancel each other out, and a cumulative score is computed for each base along the whole target sequence. Then, *findIR* searches all the pairs of positions whose cumulative scores are the same to construct candidates of perfect inverted repeats. *findIR* finally validates the candidate based on the principle that if a subsequence is a valid perfect inverted repeat, the number of nested perfect inverted repeats within the subsequence, all of which share the same center, should be equal to the half-length of the subsequence. This search and validation strategy makes *findIR* impossible to detect imperfect inverted repeats. Moreover, the prime number scoring system has its innate difficulty to represent imperfect inverted repeats that often contains a central non-palindromic spacer and/or non-complementary pairs in the pairing stem. In addition, *findIR* is limited to detect perfect inverted repeats of length shorter than 1,000 *nt*, presenting a size constraint for large genomes where longer inverted repeats may exist. Here, we developed a novel program called *detectIR* that maps the nucleotide sequence to a complex number vector so that both perfect and imperfect inverted repeats can be searched (see examples in Figure 1). Compared with *findIR*, *detectIR* adopted a totally different algorithm in perfect inverted repeat detection, which had been modified and extended to search for imperfect inverted repeats. In particular, for both perfect and imperfect inverted repeat detection, we have taken advantage of MATLAB built-in vector calculation to search and validate inverted repeats candidates of the same size simultaneously. Our program proves to be much faster and more accurate in detecting both perfect and imperfect inverted repeats than the previously mentioned tools. Moreover, our program can accept a large genome input like chromosome 1 of *Homo sapiens* and *Zea mays* that can often result in an execution crash in other tools.

Within the prime number scoring system (also called cumulative scoring system) implemented in *findIR* [24], a subsequence between two bases whose cumulative scores are identical will be recognized as a perfect inverted repeat candidate. This conclusion is based on the assumption that only the scores between reversely complementary bases can completely eliminate each other in the system. If two bases have identical score, the numbers of reversely complementary bases should be equal in the corresponding subsequence [24]. Unfortunately, this assumption is not correct for some cases, because scores also can be partly eliminated between non-complementary bases. For instance, when using the prime number mapping schema: A → 3, T → −3, G → 7, C → −7, the scores of seven nucleotides A can be eliminated out by that of three nucleotides C (same to T and G). Therefore, if two bases have an identical accumulative score, the numbers of complementary bases in the corresponding subsequence between them are not always equal. In *findIR*, such a case would be still recognized as a perfect inverted repeat candidate. Although these cases could be filtered out later by the downstream process, it clearly increases the computational workload. On the other hand, these cases can be avoided by using large prime numbers (*e.g.*, 10007, 10009), which was exactly adopted by the *findIR* [24].

Different from prime numbers, complex numbers can effectively represent both perfect and imperfect inverted repeats that contain non-complementary pairs in the stem and a central non-palindromic spacer. The complex numbers have been utilized in detecting symmetric palindromes (*e.g.*, ACGGCA, the palindromes without reverse complementary) by Gupta et al. [26], using the following mapping schema:
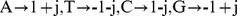



Firstly, they divided an input nucleotide sequence into subsequences with the length of a desired palindrome, and then utilized this mapping method to convert each subsequence into numerical series and rearranged the subsequence. Secondly, they use periodicity transformation to calculate a periodic sequence that is closest to the rearranged subsequence. Lastly, through calculating a coefficient between the rearranged subsequence and the periodic sequence to determine and verify each subsequence to be a valid symmetric palindrome [26]. Therefore, their method was not designed for both perfect and imperfect inverted repeat detection that requires nucleotide reverse complementary. In contrast, our usage of complex numbers is different from their approach by deploying a novel mapping schema:
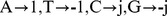



Using this mapping method, only the scores between reversely complementary bases can really cancel each other out within the numeric scoring system. This method makes sure that the numbers of reversely complementary nucleotides A and T (also C and G) are equal within the sequence candidates of perfect inverted repeat. Furthermore, we can precisely define the upper bound of the score of the imperfect inverted repeats using this complex number mapping schema - the sum of the absolute values of the real part and the imaginary part of *C* is less than or equal to *m*, 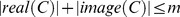 (*C* denotes the score of the subsequence, *m* represents the maximal number of mismatches allowed – the sum of all nucleotides within the central non-palindromic spacer and the non-complementary pairs in the pairing stem).

### Algorithm for perfect inverted repeat detection

The algorithm for perfect inverted repeat detection is based on the principle that a perfect inverted repeat of length *h* (*h*>4) must contain a nested perfect inverted repeat of length *h*-2, which shares the same center. So if we have obtained all the perfect inverted repeats of length *h*-2, extension of one base at both ends will derive inverted repeat candidates with length *h*. Among all these inverted repeat candidates, we then filter out those whose terminal bases are not reversely complementary.

**Figure 2:**
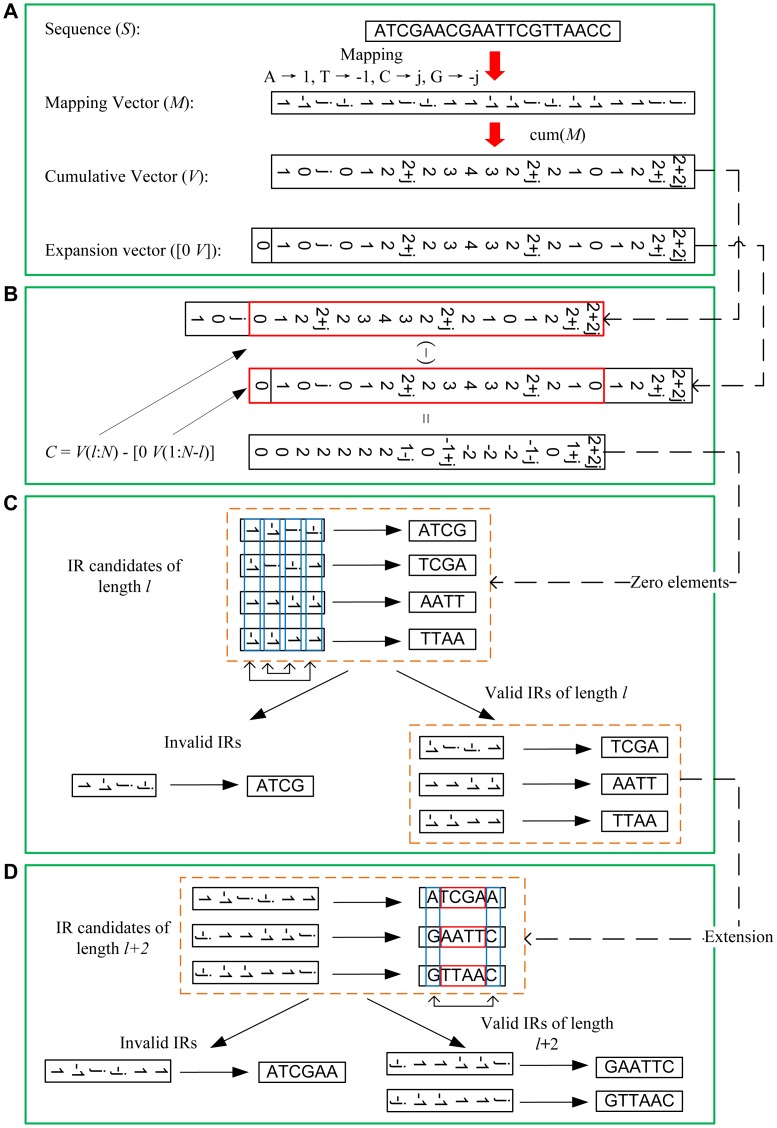
Major steps of the core algorithm for perfect inverted repeat detection. (A) Map a nucleotide sequence to a complex number vector and calculate its cumulative score value. (B) Calculate the scores of all the subsequences of length *l* (here *l* = 4, *N* is the length of input sequence). (C) Select out perfect inverted repeat candidates and determine valid perfect inverted repeats. (D) Extend one base at both ends of subsequences to obtain longer perfect inverted repeats.

**Figure 3:**
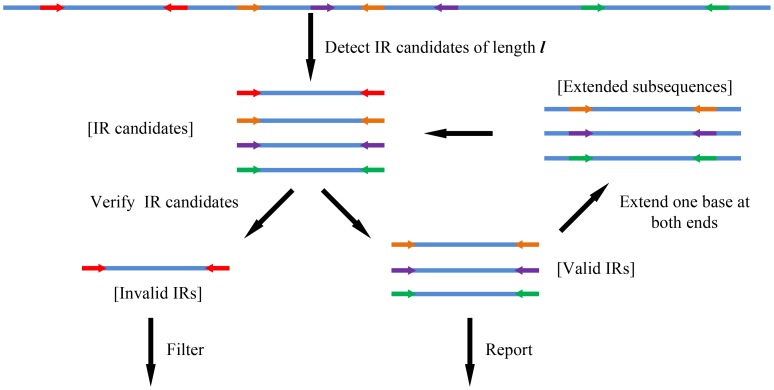
The flowchart of the core algorithm for perfect inverted repeat detection. Arrows in the same color represent the borders of inverted repeats.

The details of our algorithm are shown in Figures 2 and 3. Here, *l* is the minimal length, *L* is the maximal length of perfect inverted repeats to be detected, and *N* is the length of the input sequence *S* (*l* ≤ *L* ≤ *N*, *l* and *L* should be even number).

#### (1). Mapping sequence to vector

The input sequence *S* is mapped to a complex number vector *M*. For example, if input sequence *S* is ‘ATCGAACGAATTCGTTAACC’, *M* = [1,-1, j, -j,1, 1, j, -j, 1, 1, -1, -1, j, -j, -1, -1, 1, 1, j, j]. Then the cumulative value *V* of *M* is calculated (Figure 2A).

#### (2). Calculation of subsequence score

The scores of all the subsequences of length *l* are calculated, a subsequence score is defined as the sum of the subsequence’s corresponding vector. The scores of all the subsequences of length *l* can be obtained by the following formula,
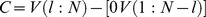



The *i*-th element *C*(*i*) represent the score of the subsequence *S*(*i*:*i*+*l*-1) = *s_i_s_i+_*_1_ *…s_i+l-_*_2_ *s_i+l-_*_1_ (Figure 2B).

#### (3). Identification of candidates

A subsequence whose score is equal to 0 will be recognized as a candidate of perfect inverted repeat. In this step we will identify all the candidates of length *l* by finding the zero elements of *C*,
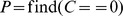



*P* represents the indices of the zero elements in vector *C*.

#### (4). Validation of candidates

For each perfect inverted repeat candidate, perfect pairing needs to be found between the front half and rear half of bases. We will keep valid perfect inverted repeat candidates, while filter out invalid cases (Figure 2C).

#### (5). Extension of inverted repeats

Base on the aforementioned principle, extend one base at both ends of inverted repeats identified in the previous step, and select out cases whose new terminal bases are reversely complementary (Figure 2D). For instance, if subsequence *S*(*i*:*i*+*h*-3) is a perfect inverted repeat of length *h*-2 identified in previous step, the extended subsequence *S*(*i*-1:*i*+*h*-2) will be a potential inverted repeat of length *h*. If its terminal bases are either G/C or A/T,
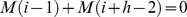



Then, *S*(*i*-1:*i*+*h*-2) is a perfect inverted repeat of length *h*.

#### (6). Repetition

Repeat the step 5 until *h* = *L*, then all the perfect inverted repeats of length ranged between *l* and *L* have been identified.

### Algorithm for imperfect inverted repeat detection

Different from the aforementioned algorithm for perfect inverted repeat detection, to find imperfect inverted repeats of length *h*, allowing maximal mismatch number *m*, which is defined as the sum of all nucleotides within the central non-palindromic spacer and the non-complementary pairs in the pairing stem, we will firstly find all subsequences of length *h*-2 with mismatch number ≤ *m*, with the assumption that the nested subsequence of an imperfect inverted repeat with mismatch number ≤ *m* must be a sequence with mismatch number ≤ *m* which share the same center. The major difference between the nested subsequence and the imperfect inverted repeat is that the terminal bases of imperfect inverted repeat must be reversely complementary (‘AAAAATTTCT’), while the former does not require meeting this condition (‘AAAATTTC’).

**Figure 4:**
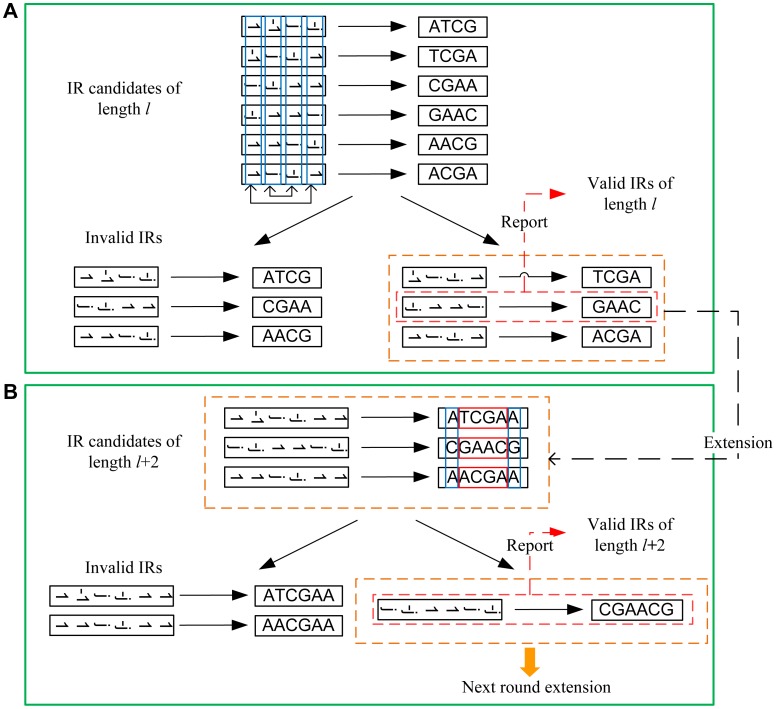
Major steps of the core algorithm for imperfect inverted repeat detection. (A) Select out imperfect inverted repeat candidates (here, we just show part of candidates), report valid imperfect inverted repeats and pick up valid subsequences for the downstream process described in the next step (here *l* = 4, *m* = 2). (B) Extend one base at both ends of subsequences to obtain longer imperfect inverted repeats. The subsequences locating at both ends of a sequence (*i.e.*, ‘ATCGAA’) should not be selected for the next round extension.

Figure 4 shows the core algorithm for imperfect inverted repeat detection. *l* is the minimal length and *L* is the maximal length of imperfect inverted repeats to be detected, and *N* is the length of the input sequence (*l* ≤ *L* ≤ *N*), *m* is the maximal number of mismatches allowing in each imperfect inverted repeat. The first two steps 1 and 2 are same to the detection of perfect inverted repeats.

#### (1). Mapping sequence to vector

#### (2). Calculation of subsequence score

#### (3). Identification of candidates

If the sum of the absolute values of the real part and the imaginary part of a subsequence’s score is less than or equal to *m*, the subsequence is recognized as an imperfect inverted repeat candidate of length *l*.
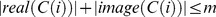



#### (4). Validation of candidates

In this step, the mismatch numbers of all the imperfect inverted repeat candidates of *length l* are calculated. Subsequences that satisfy the following three conditions: (1) the mismatch number is less than or equal to *m*; (2) the first and last base of the subsequence are reversely complementary; (3) mismatch number is not zero, will be reported as the detected imperfect inverted repeats. While, all the subsequences meeting (1) with/without (2) and (3) will be picked up for the process in the next step (see Figure 4A).

#### (5). Extension of subsequences

We will extend one nucleotide at both ends of subsequences generated in the previous step. If the two extended bases are reversely complementary, the corresponding mismatch number of the subsequence keep unchanged, otherwise the mismatch number increases by 2. Then subsequences meeting all three conditions described in step 4 are valid imperfect inverted repeats. Subsequences meeting (1) with/without (2) and (3) will be picked up for the next round extension (see Figure 4B).

For example, when subsequence *S*(*i*:*i*+*h*-3) of length *h*-2 has a mismatch number *α* less than or equal to *m*, the subsequence *S*(*i*-1:*i*+*h*-2) is a potential imperfect inverted repeats of length *h*. If,
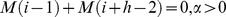



Subsequence *S*(*i*-1:*i*+*h*-2) is an imperfect inverted repeat that meets all given conditions.

#### (6). Repetition

Repeat the step 5 until *h* ***=*** *L* (or *h* ***=*** *L*-1), then all imperfect inverted repeats of odd-length (or even-length) ranged between *l* and *L* with a mismatch number ≤ *m* have been identified.

#### (7). Detection of even/odd length inverted repeats

Repeat steps 2–6, starting with calculating the score of subsequences of length *l*+1. Finally, all imperfect inverted repeats of even-length (or odd-length) ranged between *l* and *L* will been identified.

## Results

The aforementioned algorithms have been implemented into *detectIR*, which was composed of two MATLAB functions *detectPerfectIR* and *detectImperfectIR* respectively. To evaluate the performance of these two functions, they were compared with four inverted repeat detection tools BioPHP (A PHP program obtained from http://www.biophp.org/minitools/find_palindromes), EMBOSS *palindrome* tool (Stable version 6.3.1), MATLAB built-in *palindromes* function (Matlab R2013b) and MATLAB-based *findIR*. HIV-1 genome and chromosome 1 from *Arabidopsis thaliana*, *Homo sapiens* and *Zea mays* were used as the test data. The detailed step-by-step guide for testing and comparison is available on our sourceforge project website (https://sourceforge.net/projects/detectir). Our source codes and relevant documents are also available as in File S1. The result comparison of perfect inverted repeat detection and imperfect inverted repeat detection is presented below separately. For smaller genomes like *Arabidopsis thaliana* and HIV-1, a standard workstation with 8G RAM should be enough for testing, while large genomes like *Homo sapiens* and *Zea mays* will require at least 32G RAM. For the test results described below, all the tests were performed using Ubuntu 12.04 (precise) 64-bit platform with Intel Xeon (2.00 GHz) processer and 125.9 GB RAM.

### Perfect inverted repeat detection

Here, we used each program to search perfect inverted repeats with length between 10 and 1000 *nt*. The detection results are summarized in Table 1. The length distributions of the perfect inverted repeats detected by *detectIR* are shown in Figure S1. It is clear that the numbers of perfect inverted repeats decrease with the increase of inverted repeat length, shorter inverted repeats are much more abundant in genomes (see Figure S1).

**Table 1:** The comparison of *detectPerfectIR* with BioPHP, EMBOSS, MATLAB and *findIR* for perfect inverted repeat detection.

	HIV-1		Arabidopsis thaliana		Homo sapiens		Zea mays	
	IR count	runtime	IR count	runtime	IR count	runtime	IR count	runtime
BioPHP	7	5.56 m	*	*	*	*	*	*
EMBOSS(*palindrome*)	5	<1 s	61,705	34.53 m	347,688	38.74 h	448,928	4.28 h
MATLAB(*palindromes*)	13	0.06 s	266,187	3.27 m	*	*	*	*
*findIR*	7	0.23 s	142,249	8.80 m	746,925	1.16 h	1,147,428	1.51 h
*detectPerfectIR*	7	0.05 s	142,249	11.85 s	746,925	1.72 m	1,147,428	2.00 m

*Program crashed in execution or output nothing after 10-day execution.

To determine the accuracy of *detectPerfectIR*, the outputs from different programs using the same input data were compared against *detectPerfectIR* respectively. The differences discovered between them can be classified into two categories (1) entries only present in the output of *detectPerfectIR*, (2) entries only present in the output of BioPHP, EMBOSS (*palindrome*), MATLAB (*palindromes*) or *findIR*. As shown in Table 1, consequently, the outputs of *detectPerfectIR* and *findIR* are identical, while *detectPerfectIR* performs much more efficiently.

#### Entries only present in the output of detectPerfectIR and findIR

The entries only detected by *detectPerfectIR* and *findIR* were found to be valid perfect inverted repeats by human validation. In other words, these cases are missed by the other compared tools. For HIV-1 genome, both EMBOSS and MATLAB missed 2 perfect inverted repeats (see File S2 and S3). For chromosome 1 of *Arabidopsis thaliana*, EMBOSS missed 80,548 perfect inverted repeats (see File S4) and MATLAB missed 86,759 perfect inverted repeats (see File S5). For chromosome 1 of *Homo sapiens*, EMBOSS missed 399,237 perfect inverted repeats (see File S6). For chromosome 1 of *Zea mays*, EMBOSS missed 703,018 perfect inverted repeats (see File S7).

#### Entries only present in the output of the other compared tools

The entries only found by the other tools prove to be invalid perfect inverted repeats by human validation, which mean the outputs of these tools contain false positives. For the HIV-1 genome, the output of MATLAB contains 8 cases with a gap in the center, like ‘CAAAAATTTTG’ (see File S3). For chromosome 1 of *Arabidopsis thaliana*, the output of EMBOSS contains 4 invalid cases like ‘A-N(60)-T’ etc (see File S4). And the output of MATLAB contains 210,697 invalid cases, 46915 of them are subsequences like ‘ATTTTTTAAAAAT’ with a gap in the center and 163,782 of them are subsequences like ‘NNNNNNNNNN’ or ‘A-N(27)-T’ (see File S5). For chromosome 1 of *Zea mays*, the output of EMBOSS contains 4,518 invalid cases (see File S7).

### Imperfect inverted repeat detection

In imperfect inverted repeat detection, some tools define continuous mismatches in the center of inverted repeat as gaps; mismatches in other locations are still considered as mismatches, while our algorithm does not differentiate these two types of mismatch. For example, EMBOSS *palindrome* tool will recognize sequence ‘AACAACTTTCTT’ as an inverted repeat with 1 mismatch and 2 gaps, our tool will recognize it as an inverted repeat with 4 mismatches. MATLAB *palindromes* function can only deal with imperfect inverted repeats containing gaps. So, here we let our function *detectImperfectIR* and MATLAB *palindromes* function detect imperfect inverted repeats of length between 20 and 1000 *nt*, mismatch number (or gap number) ≤6, and let EMBOSS palindrome tool search imperfect inverted repeats of length between 20 and 1000 *nt*, gap number ≤2, and mismatch number ≤2 (Using the definition of EMBOSS). BioPHP and *findIR* are not used here, because they are designed to detect only the perfect inverted repeats. Summaries of the detection results are shown in Table 2. The length distributions of the imperfect inverted repeats detected by *detectIR* are shown in Figure S2. Obviously, the length distribution of imperfect inverted repeats is similar to that of perfect inverted repeats. The number of imperfect inverted repeats of even length is more than those of odd length, and the underlying reason may be that the odd-length inverted repeats must contain a spacer in the middle, which means that the mismatches in the stem should be less. To test this assumption, we run detectIR with an odd maximum mismatch number (*m* = 7). As shown in Figure S3, the numbers of odd-length imperfect inverted repeats are approximate to the numbers of even-length imperfect inverted repeats.

**Table 2:** The comparison of *detectImperfectIR* with EMBOSS and MATLAB for imperfect inverted repeat detection.

	HIV-1		Arabidopsis thaliana		Homo sapiens		Zea mays	
	IR count	runtime	IR count	runtime	IR count	runtime	IR count	runtime
EMBOSS(*palindrome*)	8	<1 s	65,596	43.48 m	429,662	39.96 h	619,889	5.91 h
MATLAB(*palindromes*)	1	0.07 s	173,659	11.00 m	*	*	*	*
*detectImperfectIR*	37	0.09 s	311,369	1.48 m	2,166,638	11.83 m	2,878,686	16.63 m

*Program crashed in execution or output nothing after 10-day execution.

In order to make an unbiased comparison, we will filter out entries with a mismatch number larger than 2 or a gap number larger than 2 (Using the definition of EMBOSS) in the output of *detectImperfectIR* before comparing it with the output of EMBOSS.

#### Entries only present in the output of detectImperfectIR

All these entries are validated by human to be imperfect inverted repeats that meet the defined criteria. For HIV-1, EMBOSS missed 9 cases (see File S8), and MATLAB missed 36 cases (see File S9). For instance, both EMBOSS and MATLAB missed case ‘ATCAGATGCTAAAGCATATGAT’. For chromosome 1 of *Arabidopsis thaliana*, EMBOSS missed 78,293 cases (see File S10) and MATLAB missed 304,394 cases (see File S11). Both EMBOSS and MATLAB missed cases like ‘CTTTAGCATTTATTCTGAAG’ and ‘ATAATTTAAAATAAAATTAT’. For chromosome 1 of *Homo sapiens*, EMBOSS missed 618,357 cases (see File S12). For chromosome 1 of *Zea mays*, EMBOSS missed 825,455 cases (see File S13).

#### Entries only present in the output of the other compared tools

The entries only present in the output of the other compared tools prove to be false positives again. For chromosome 1 of *Arabidopsis thaliana*, the output of EMBOSS contains 1,765 perfect inverted repeats and 23 entries like ‘A-N(27)-TT’ (see File S10). The output of MATLAB contains 3,091 perfect inverted repeats and 163,593 entries like ‘N(20)’ or ‘A-N(27)-T’ (see File S11). For chromosome 1 of *Homo sapiens*, the output of EMBOSS contains 6,771 perfect inverted repeats (see File S12). For chromosome 1 of *Zea mays*, the output of EMBOSS contains 17,926 perfect inverted repeats and 27,761 entries like ‘A-N(100)-T’ (see File S13).

### Random nucleotide sequence test

**Figure 5:**
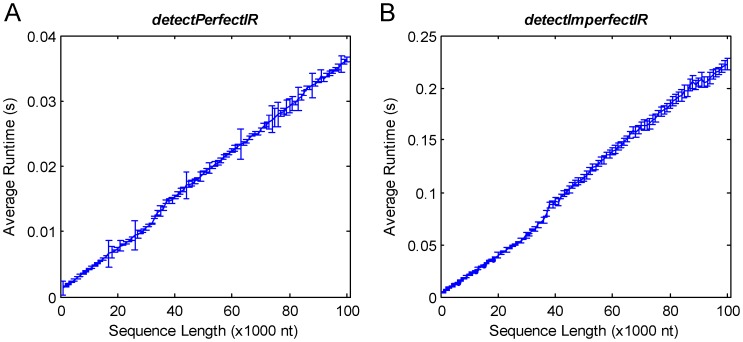
Average runtimes of *detectPerfectIR* and *detectImperfectIR* using random nucleotide sequence inputs of varied lengths. (A) The average runtimes of *detectPerfectIR* using random nucleotide sequence inputs of varied lengths. (B) The average runtimes of *detectImperfectIR* using random nucleotide sequence inputs of varied lengths.

To evaluate the efficiency of *detectIR*, we generated several random nucleotide sequences of varied lengths, use function *detectPerfectIR* to detect perfect inverted repeats of length between 4 and 1000, and use function *detectImperfectIR* to detect imperfect inverted repeats of length between 10 and 1000 with mismatches less than or equal to 6. The average runtimes are shown below in Figure 5.

As shown in Figure 5, average runtime of the *detectIR* increases with the increase of the sequence length. The execution time of *detectImperfectIR* is ∼6 fold of that of *detectPerfectIR* with same input data, because the perfect inverted repeats has more strict requirements than imperfect inverted repeats which reduce the search space. So it is clear that *detectIR* shows good scalability dealing with large genome sequence inputs.

## Availability and Future Directions

*detectIR* is platform independent and can be used in Windows or Linux as long as MATLAB can be run. The source codes, test and comparison scripts, and documents are freely available at: https://sourceforge.net/projects/detectir.

Clearly, inverted repeats are not randomly distributed in both prokaryotic and eukaryotic genomes. Without a doubt, more wet-lab experiments for important genes are needed to clearly understand their biological functions. However, *in silico* genome-wide scan of inverted repeats can effectively help us to determine their overall distributions and characteristics and discover the groups of inverted repeats that are more likely to have important biological functions. As shown in Information S1, we used *detectIR* to conduct comparative study of perfect and imperfect inverted repeats in *Arabidopsis* genome. We found that both perfect and imperfect inverted repeats are not randomly distributed along the genome, and imperfect inverted repeats are much more abundant than perfect inverted repeats. In particular, imperfect inverted repeats are significantly enriched in near intergenic regions than far intergenic regions, while both perfect and imperfect inverted repeats are significantly more abundant in introns than exons. Our results are in line with the findings in human and yeast genomes [3] [22] [23]. Obviously, the inverted repeats in introns and promoter regions are worthy of closer examination in the future.

In conclusion, we developed an accurate and efficient program *detectIR* for detecting both perfect and imperfect inverted repeats in a given nucleotide sequence. *detectIR* is capable of processing large genome sequences, given enough memory in computation. Compared to BioPHP, EMBOSS *palindrome* tool, MATLAB built-in *palindromes* function and MATLAB-based *findIR*, the test results show that our program can more efficiently detect inverted repeats without sacrificing accuracy. Future directions will focus on improving the program to detect imperfect inverted repeats with indels within the paring stem, and reducing the memory consumption of program.

## Supporting Information

Figure S1 **Length distributions of perfect inverted repeats in different species detected by** ***detectIR*** **.**
(DOC)

Figure S2 **Length distributions of imperfect inverted repeats in different species detected by** ***detectIR*** **with the maximum mismatch number of 6 (** ***m*** **= 6).**
(DOC)

Figure S3 **Length distributions of imperfect inverted repeats in different species detected by** ***detectIR*** **with the maximum mismatch number of 7 (** ***m*** **= 7).**
(DOC)

File S1 **Source codes, test and comparison scripts, and user documents of** ***detectIR*** **.**
(TGZ)

File S2 **The output files of the perfect inverted repeats detected differentially by** ***detectIR*** **and EMBOSS** ***palindrome*** **tool (Using the parameters described in the manuscript and HIV-1 genome as input data).**
(TGZ)

File S3 **The output files of the perfect inverted repeats detected differentially by** ***detectIR*** **and MATLAB** ***palindromes*** **function (Using the parameters described in the manuscript and HIV-1 genome as input data).**
(TGZ)

File S4 **The output files of the perfect inverted repeats detected differentially by** ***detectIR*** **and EMBOSS** ***palindrome*** **tool (Using the parameters described in the manuscript and chromosome 1 of** ***Arabidopsis thaliana*** **as input data).**
(TGZ)

File S5 **The output files of the perfect inverted repeats detected differentially by** ***detectIR*** **and MATLAB** ***palindromes*** **function (Using the parameters described in the manuscript and chromosome 1 of** ***Arabidopsis thaliana*** **as input data).**
(TGZ)

File S6 **The output files of the perfect inverted repeats detected differentially by** ***detectIR*** **and EMBOSS** ***palindrome*** **tool (Using the parameters described in the manuscript and chromosome 1 of** ***Homo sapiens*** **as input data).**
(TGZ)

File S7 **The output files of the perfect inverted repeats detected differentially by** ***detectIR*** **and EMBOSS** ***palindrome*** **tool (Using the parameters described in the manuscript and chromosome 1 of** ***Zea mays*** **as input data).**
(TGZ)

File S8 **The output files of the imperfect inverted repeats detected differentially by** ***detectIR*** **and EMBOSS** ***palindrome*** **tool (Using the parameters described in the manuscript and HIV-1 genome as input data).**
(TGZ)

File S9 **The output files of the imperfect inverted repeats detected differentially by** ***detectIR*** **and MATLAB** ***palindromes*** **function (Using the parameters described in the manuscript and HIV-1 genome as input data).**
(TGZ)

File S10 **The output files of the imperfect inverted repeats detected differentially by** ***detectIR*** **and EMBOSS** ***palindrome*** **tool (Using the parameters described in the manuscript and chromosome 1 of** ***Arabidopsis thaliana*** **as input data).**
(TGZ)

File S11 **The output files of the imperfect inverted repeats detected differentially by** ***detectIR*** **and MATLAB** ***palindromes*** **function (Using the parameters described in the manuscript and chromosome 1 of** ***Arabidopsis thaliana*** **as input data).**
(TGZ)

File S12 **The output files of the imperfect inverted repeats detected differentially by** ***detectIR*** **and EMBOSS** ***palindrome*** **tool (Using the parameters described in the manuscript and chromosome 1 of** ***Homo sapiens*** **as input data).**
(TGZ)

File S13 **The output files of the imperfect inverted repeats detected differentially by** ***detectIR*** **and EMBOSS** ***palindrome*** **tool (Using the parameters described in the manuscript and chromosome 1 of** ***Zea mays*** **as input data).**
(TGZ)

Information S1 **The genome-wide distribution of perfect and imperfect inverted repeats in** ***Arabidopsis thaliana*** **.**
(DOC)
