# Supplementary material for: detectIR: A Novel Program for Detecting Perfect and Imperfect Inverted Repeats Using Complex Numbers and Vector Calculation
Source: PLoS One. 2014 Nov 19;9(11):e113349. doi: 10.1371/journal.pone.0113349 (PMC4237412; doi:10.1371/journal.pone.0113349)
Supplement: Figure S1 — Length distributions of perfect inverted repeats in different species detected by detectIR . (DOC) [file pone.0113349.s001.doc]

**A B**

**C D**

**Figure S1. Length distributions of perfect inverted repeats in different species detected by *detectIR***

For Homo sapiens and Zea mays, some perfect inverted repeats are over 100 *nt* in length. (A) The length distribution of perfect inverted repeats detected in HIV-1 genome. (B) The length distribution of perfect inverted repeats detected in chromosome 1 of *Arabidopsis thaliana*. (C) The length distribution of perfect inverted repeats detected in chromosome 1 of *Homo sapiens*. (D) The length distribution of perfect inverted repeats detected in chromosome 1 of *Zea mays*.
