# Supplementary material for: detectIR: A Novel Program for Detecting Perfect and Imperfect Inverted Repeats Using Complex Numbers and Vector Calculation
Source: PLoS One. 2014 Nov 19;9(11):e113349. doi: 10.1371/journal.pone.0113349 (PMC4237412; doi:10.1371/journal.pone.0113349)
Supplement: Figure S2 — Length distributions of imperfect inverted repeats in different species detected by detectIR with the maximum mismatch number of 6 ( m = 6). (DOC) [file pone.0113349.s002.doc]

**A B**

**C D**

**Figure S2. Length distributions of imperfect inverted repeats in different species detected by *detectIR* with the maximum mismatch number of 6 (*m*=6)**

For Homo sapiens and Zea mays, some imperfect inverted repeats are over 100 *nt* in length. Blue bars represent the imperfect inverted repeats of even length and red bars represent the imperfect inverted repeats of odd length. (A) The length distribution of imperfect inverted repeats detected in HIV-1 genome. (B) The length distribution of imperfect inverted repeats detected in chromosome 1 of *Arabidopsis thaliana*. (C) The length distribution of imperfect inverted repeats detected in chromosome 1 of *Homo sapiens*. (D) The length distribution of imperfect inverted repeats detected in chromosome 1 of *Zea mays*.
