# Supplementary material for: detectIR: A Novel Program for Detecting Perfect and Imperfect Inverted Repeats Using Complex Numbers and Vector Calculation
Source: PLoS One. 2014 Nov 19;9(11):e113349. doi: 10.1371/journal.pone.0113349 (PMC4237412; doi:10.1371/journal.pone.0113349)
Supplement: Information S1 — The genome-wide distribution of perfect and imperfect inverted repeats in Arabidopsis thaliana . (DOC) [file pone.0113349.s017.doc]

**The genome-wide distribution of perfect and imperfect inverted repeats in *Arabidopsis thaliana***

**Introduction**

There are five nuclear chromosomes and two organelle chromosomes in *Arabidopsis thaliana.* We used TAIR10 genome sequence and gene annotation for our data analysis (ftp://ftp.arabidopsis.org/home/tair/Genes/TAIR10_genome_release/). Our tool *detectIR* was adopted to detect perfect and imperfect inverted repeats in *Arabidopsis thaliana* genome. We detected perfect inverted repeats of length between 10 and 1000 *nt*, and identified imperfect inverted repeats of length between 20 and 1000 *nt* with the maximum mismatches of 6 *nt*, which include both the uncomplimentary nucleotides in the pairing stem and the nucleotides within the central non-palindromic spacer. The distributions of perfect and imperfect inverted repeats among different intragenic (*i.e.*, genes, exons, introns, 5' UTRs, 3'-UTRs, CDS regions) and intergenic regions (*i.e.*, far intergenic versus near intergenic regions) were analyzed. The data analyses show that imperfect inverted repeats are much more abundant than perfect ones in *Arabidopsis* genome, and both perfect and imperfect inverted repeats are not randomly distributed within the genome.

As shown in Figure S1.1, the intergenic regions were defined as those without any annotated gene, either protein coding gene or non-coding gene, in both strands. The parts of intergenic regions which are at least 10,000 *nt* away from the flanking gene ends were considered to be far intergenic regions, while 200 *nt* intergenic regions flanking gene ends were considered to be near intergenic regions. The genomic region coordinates were obtained and determined using TAIR10 GFF file. Inverted repeat base enrichments among different intragenic and intergenic regions were compared and tested using the Chi-square test, at 0.95 confidence level.

**Result**

*Arabidopsis thaliana* genome totally has 119,667,750 base pairs (see Figure S1.2). Our genome-wide inverted repeat detection has detected 574,606 perfect inverted repeats and 1,237,109 imperfect inverted repeats (see Figure S1.3). We found that imperfect inverted repeats are much more abundant than perfect inverted repeats in *Arabidopsis thaliana* genomes.

The intragenic and intergenic distribution of imperfect inverted repeats is shown in Figure S1.4 while the intragenic and intergenic distribution of perfect inverted repeats is shown in Figure S1.5. The total nucleotide bases, the total nucleotide bases of imperfect inverted repeats, and the total nucleotide bases of perfect inverted repeats among different intragenic and intergenic regions are shown in Figure S1.6, S1.7 and S1.8 respectively. The percentage of imperfect inverted repeat bases in different genomic regions is shown in Figure S1.9. The percentage of perfect inverted repeat bases in different genomic regions is shown in Figure S1.10. The comparison between the percentage of imperfect inverted repeat bases and the percentage of perfect inverted repeat bases in different genomic regions is shown in Figure S1.11.

Significantly higher perfect and imperfect inverted repeat abundance was observed in the intergenic regions in comparison with the gene regions (p-value < 0.0001), suggesting inverted repeat enrichment in the intergenic regions. Such a lower presentation of inverted repeats in gene regions might contribute to genomic stability because inverted repeat sequences are known sources of genetic instability [1][2].

Within the genic regions, both perfect and imperfect inverted repeat bases were significantly enriched in introns in comparison to exons (p-value < 0.0001). This higher abundance of inverted repeats in introns might be due to that fact that introns contain many *cis*-regulator elements. Inverted repeats could act as a regulatory element that involves in biological processes such as alternative splicing of pre-mRNAs in eukaryotes [3][4][5].

In comparison with the far intergenic region, near intergenic region has higher imperfect inverted repeat (p-value < 0.0001). Inverted repeats were found most frequently in promoter regions near the transcriptional start sites, indicating the potential function of inverted repeats in transcriptional regulation [6][7]. Our results seem to support this observation.

**Reference**

1. Gordenin DA, Lobachev KS, Degtyareva NP, Malkova AL, Perkins E, et al. (1993) Inverted DNA repeats: a source of eukaryotic genomic instability. Mol Cell Biol 13: 5315–5322.

2. Brázda V, Laister RC, Jagelská EB, Arrowsmith C (2011) Cruciform structures are a common DNA feature important for regulating biological processes. BMC Mol Biol 12: 33.

3. Nasim F-UH, Hutchison S, Cordeau M, Chabot B (2002) High-affinity hnRNP A1 binding sites and duplex-forming inverted repeats have similar effects on 5’splice site selection in support of a common looping out and repression mechanism. Rna 8: 1078–1089.

4. Martinez-Contreras R, Fisette J-F, Nasim FH, Madden R, Cordeau M, et al. (2006) Intronic Binding Sites for hnRNP A/B and hnRNP F/H Proteins Stimulate Pre-mRNA Splicing. PLoS Biol 4: e21. doi:10.1371/journal.pbio.0040021.

5. Howe KJ, Ares M (1997) Intron self-complementarity enforces exon inclusion in a yeast pre-mRNA. Proc Natl Acad Sci 94: 12467–12472.

6. Lu L, Jia H, Dröge P, Li J (2007) The human genome-wide distribution of DNA palindromes. Funct Integr Genomics 7: 221–227. doi:10.1007/s10142-007-0047-6.

7. Humphrey-Dixon EL, Sharp R, Schuckers M, Lock R, Gulick P (2011) Comparative genome analysis suggests characteristics of yeast inverted repeats that are important for transcriptional activity. Genome 54: 934–942. doi:10.1139/g11-058.

**Figures**

**Figure S1.1 Diagram of different intragenic and intergenic regions.**

(A) Far intergenic region is a part of intergenic region which is 10,000 bp away from the flanking gene ends. (B) Near intergenic region is defined as the 200 bp, upstream of the 5' gene end or downstream of the 3' gene end.


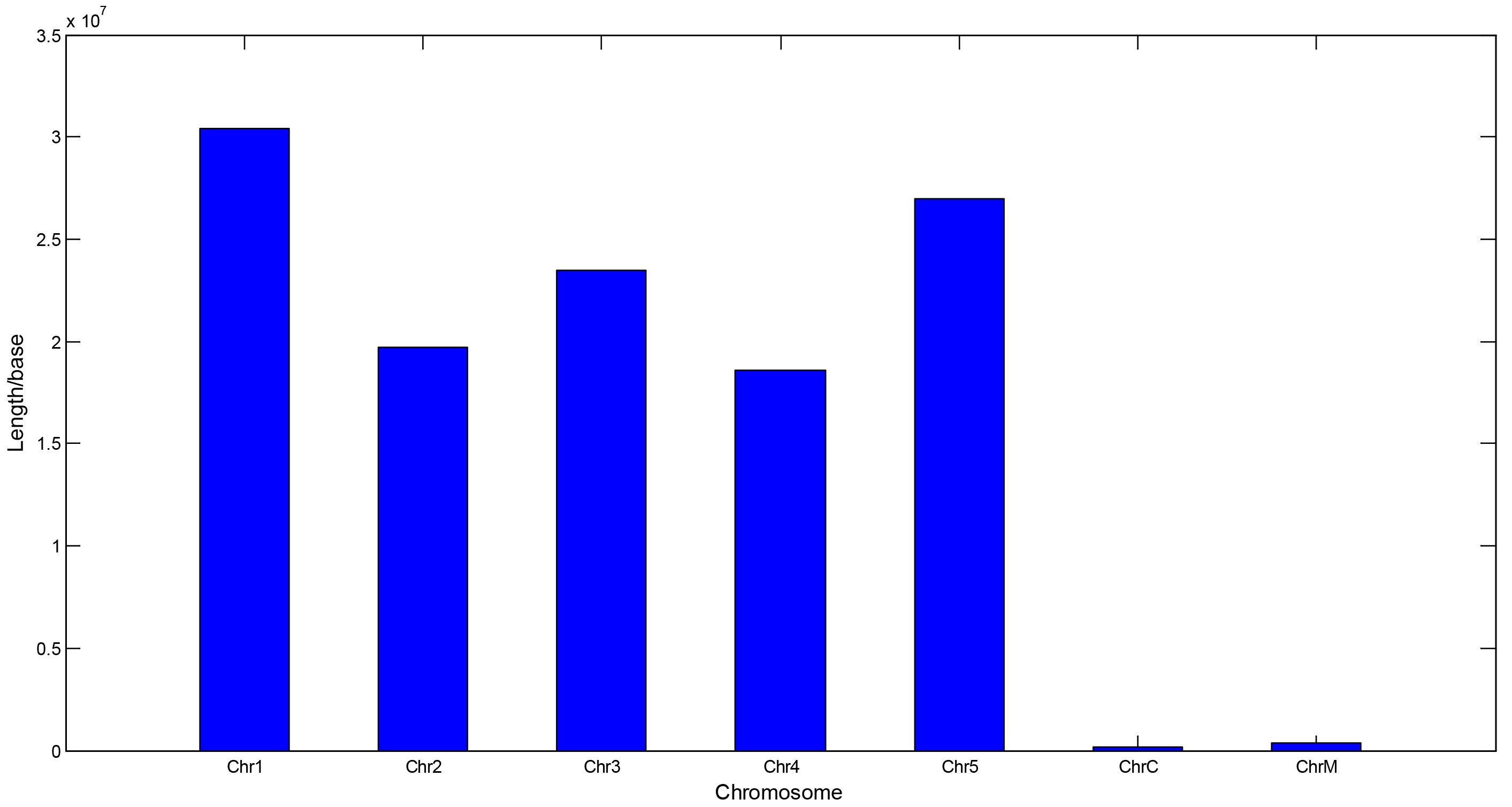


**Figure S1.2 Chromosome lengths of *Arabidopsis thaliana*.**


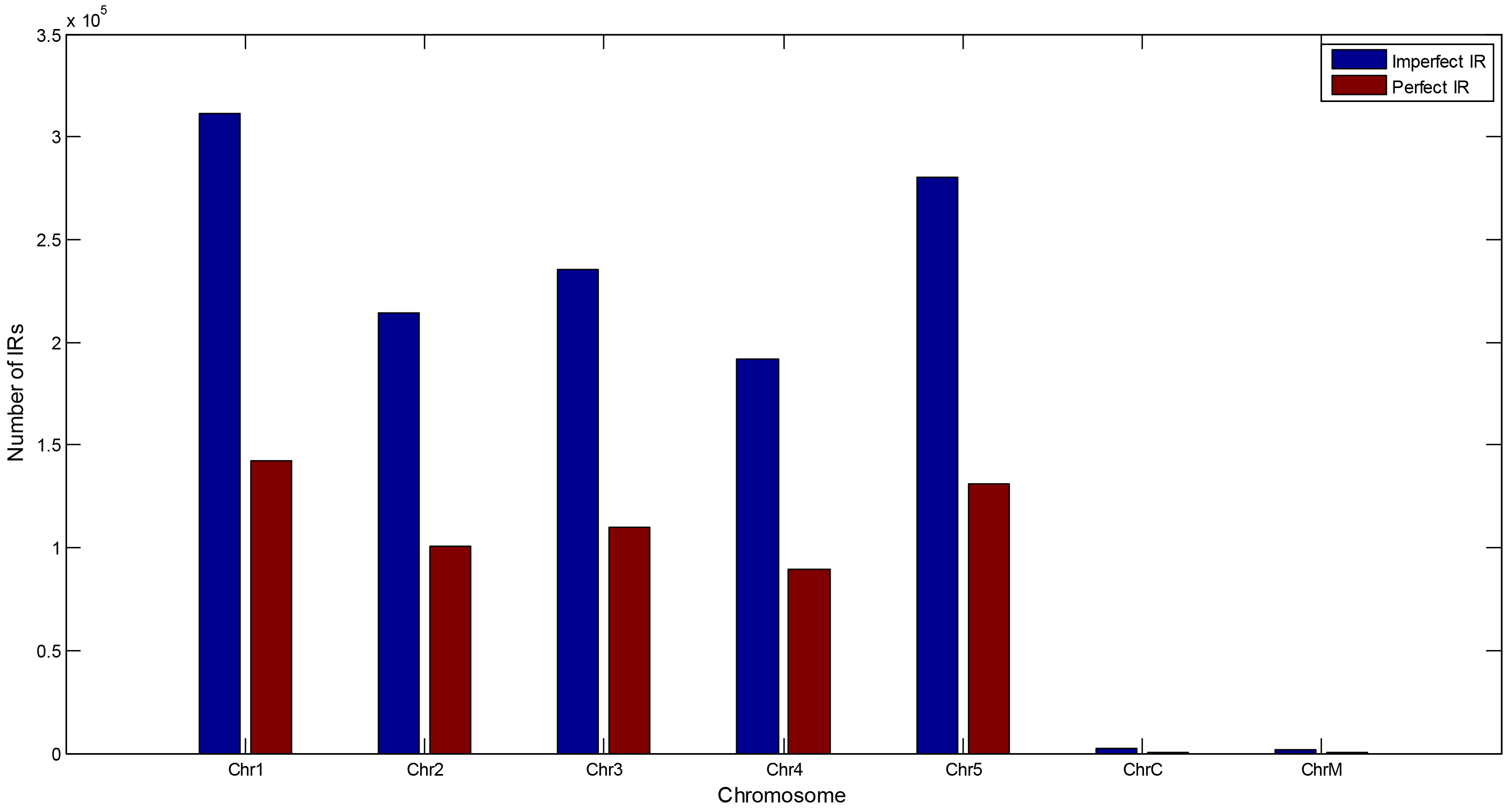


**Figure S1.3 Abundance of imperfect and perfect inverted repeats detected in chromosomes of *Arabidopsis thaliana*.**


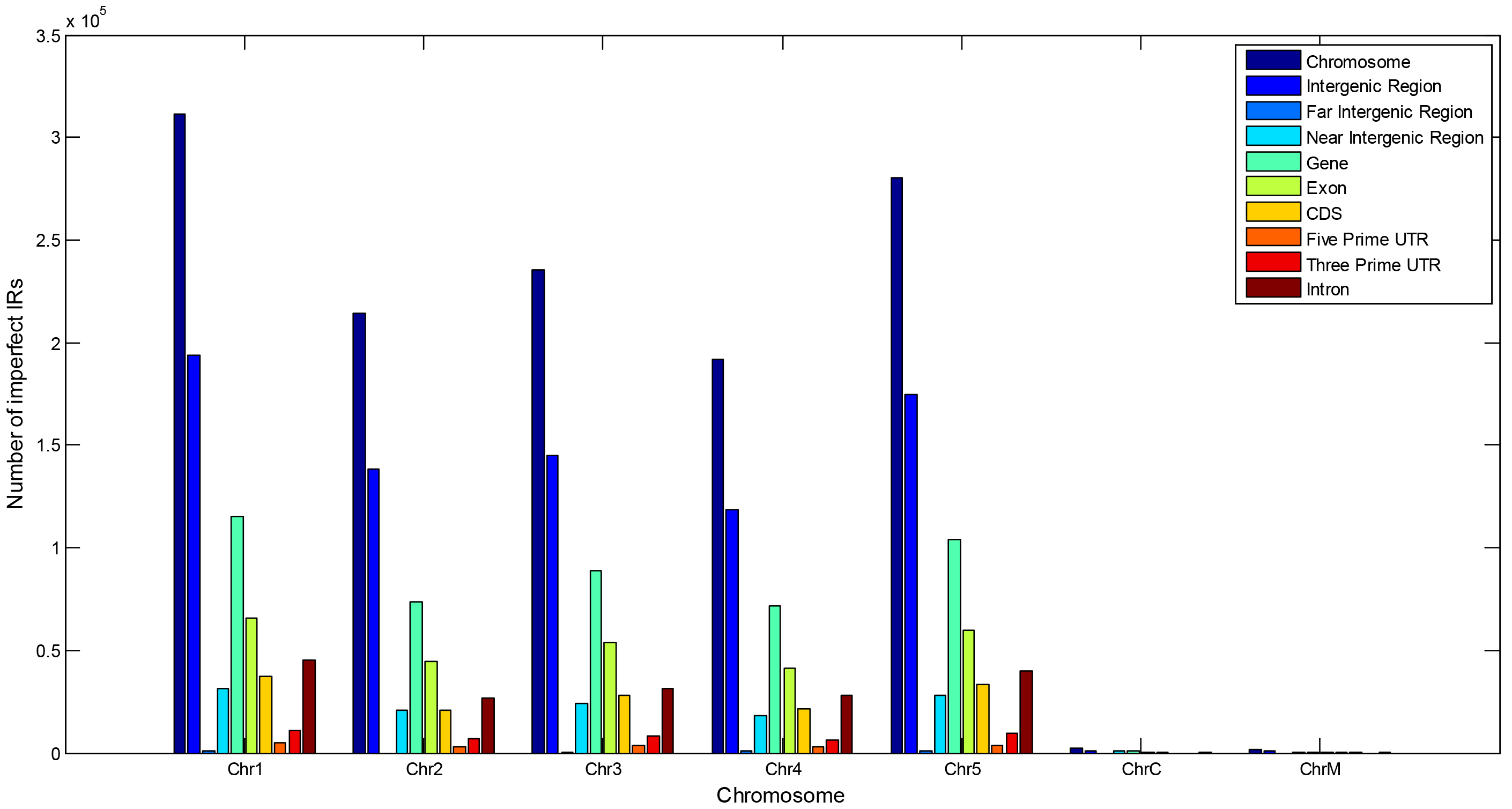


**Figure S1.4 The intragenic and intergenic distribution of imperfect inverted repeats detected in chromosomes of *Arabidopsis thaliana*.**


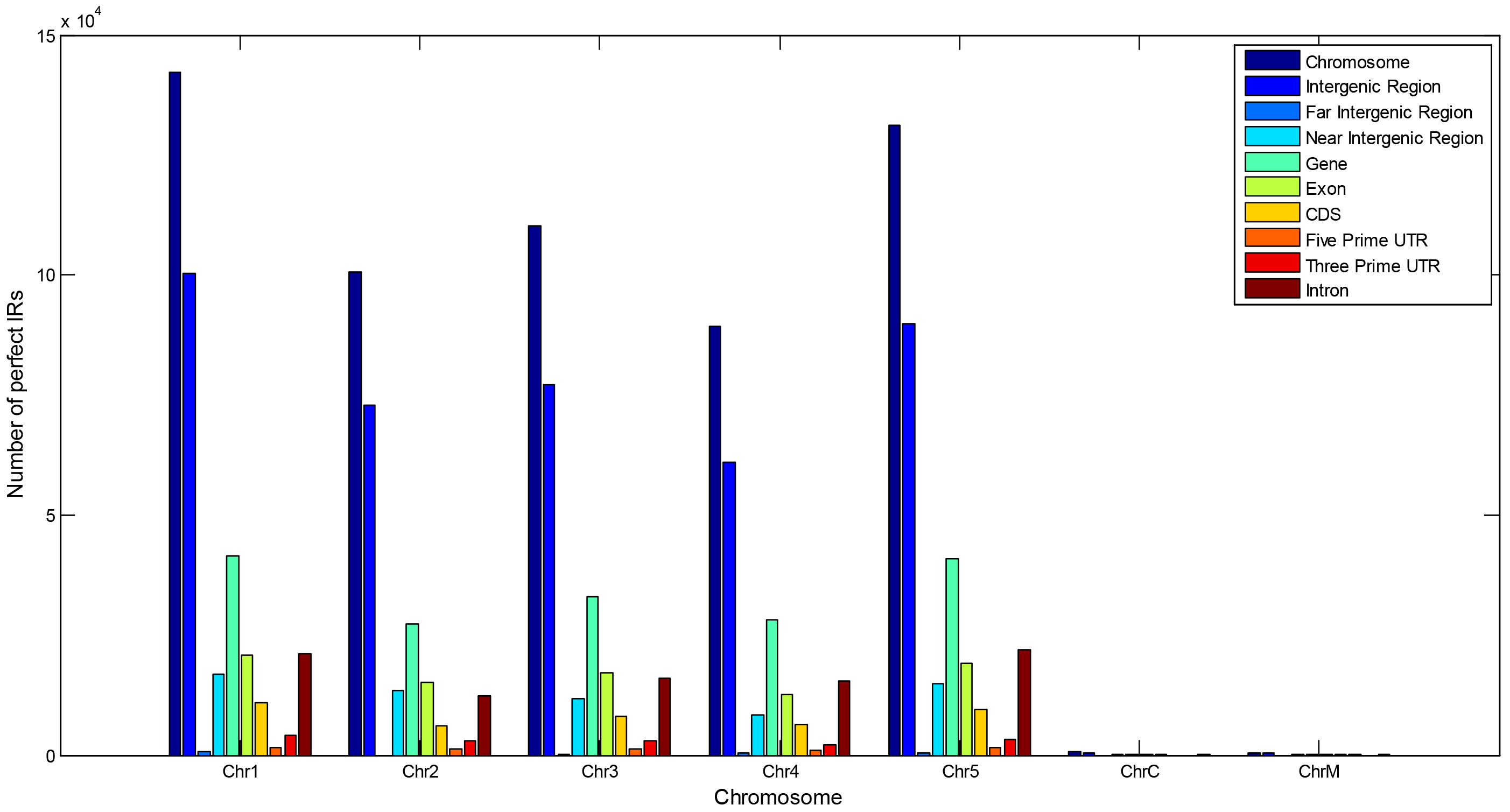


**Figure S1.5 The intragenic and intergenic distribution of perfect inverted repeats detected in chromosomes of *Arabidopsis thaliana*.**


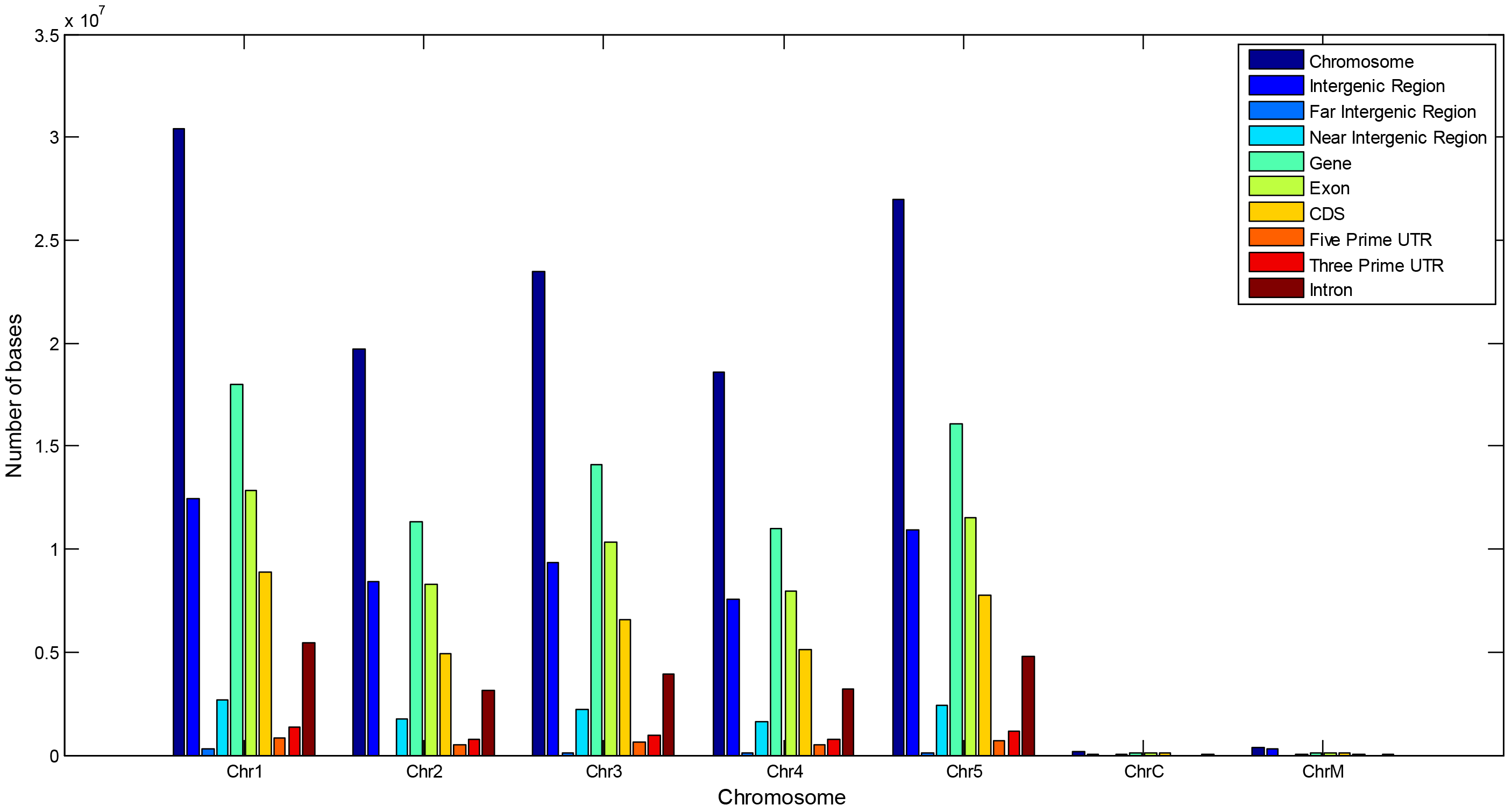


**Figure S1.6 The total nucleotide bases of different intragenic and intergenic regions.**


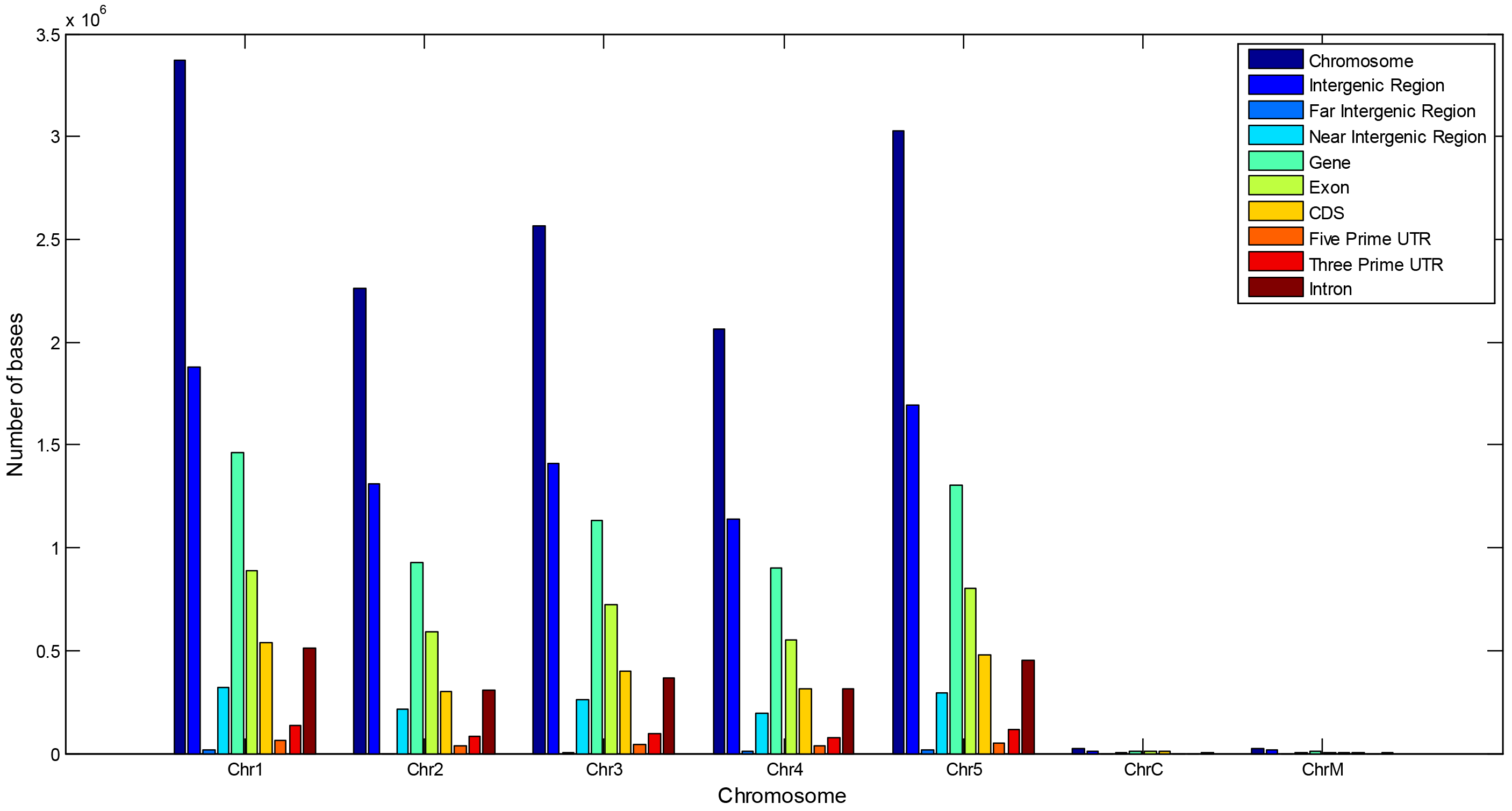


**Figure S1.7 The total nucleotide bases of imperfect inverted repeats among various genomic regions.**


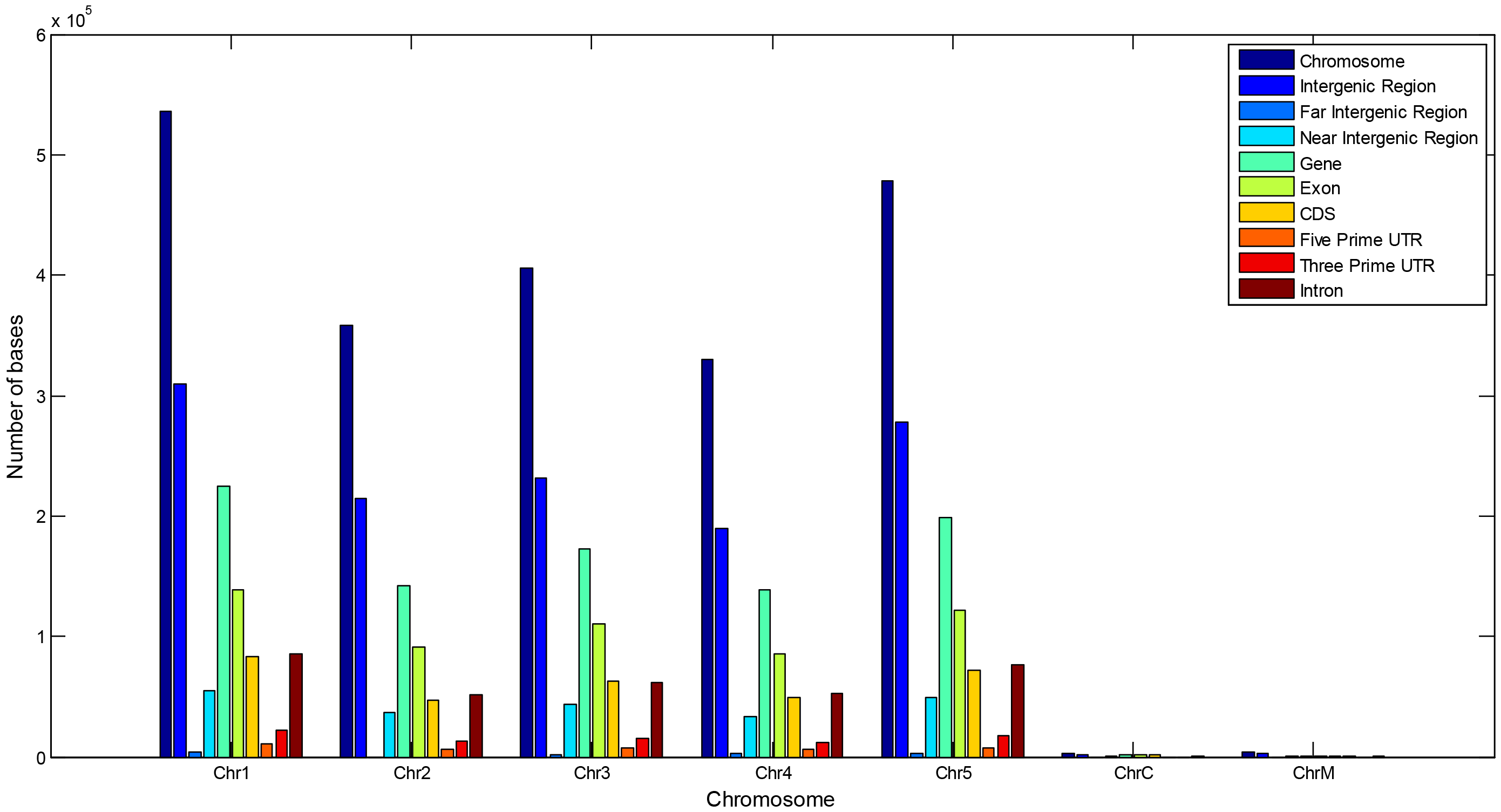


**Figure S1.8 The total nucleotide bases of perfect inverted repeats among various genomic regions.**


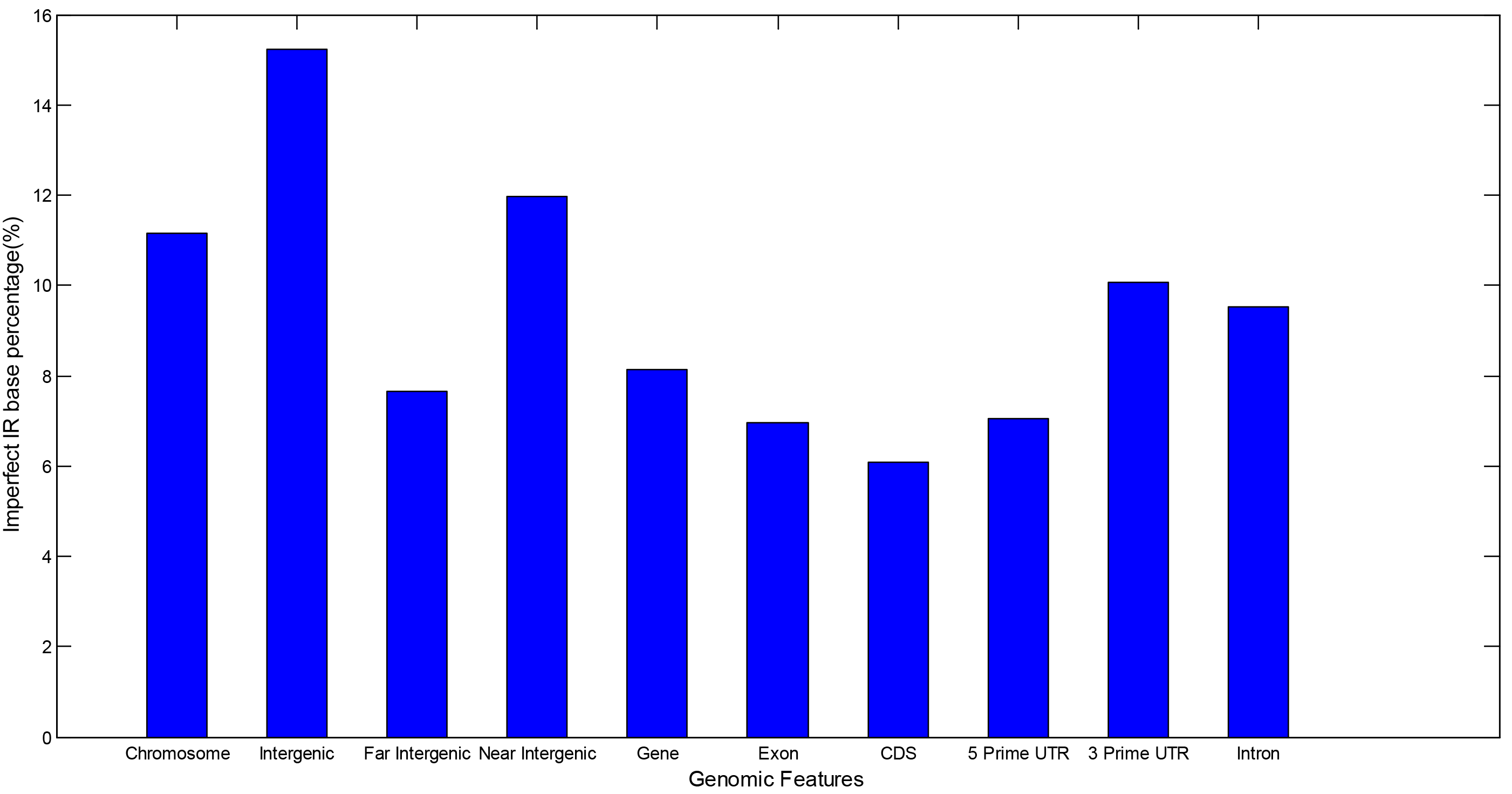


**Figure S1.9 The percentage of imperfect inverted repeat bases within various genomic regions across all chromosomes.**


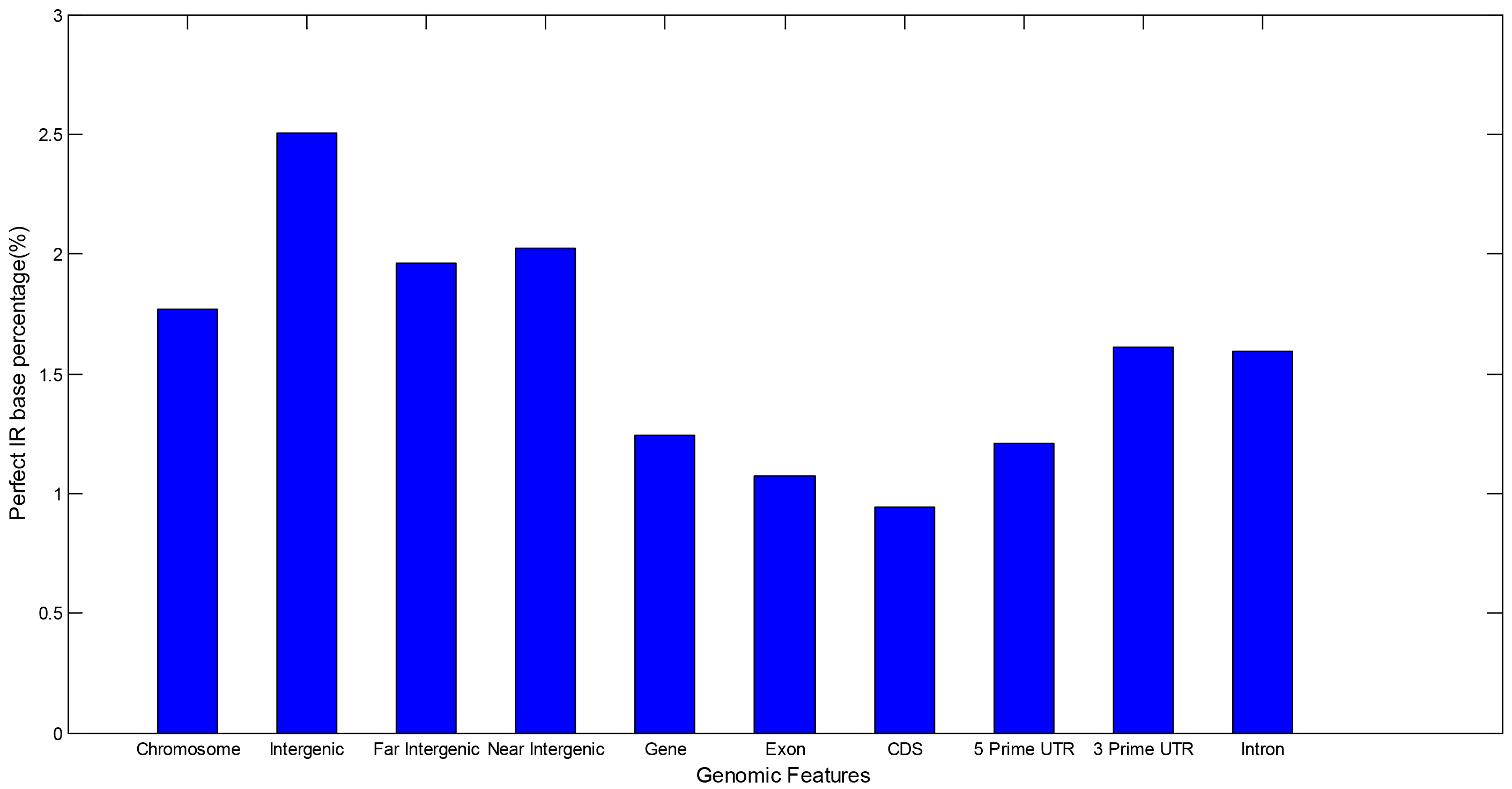


**Figure S1.10 The percentage of perfect inverted repeat bases within various genomic regions across all chromosomes.**


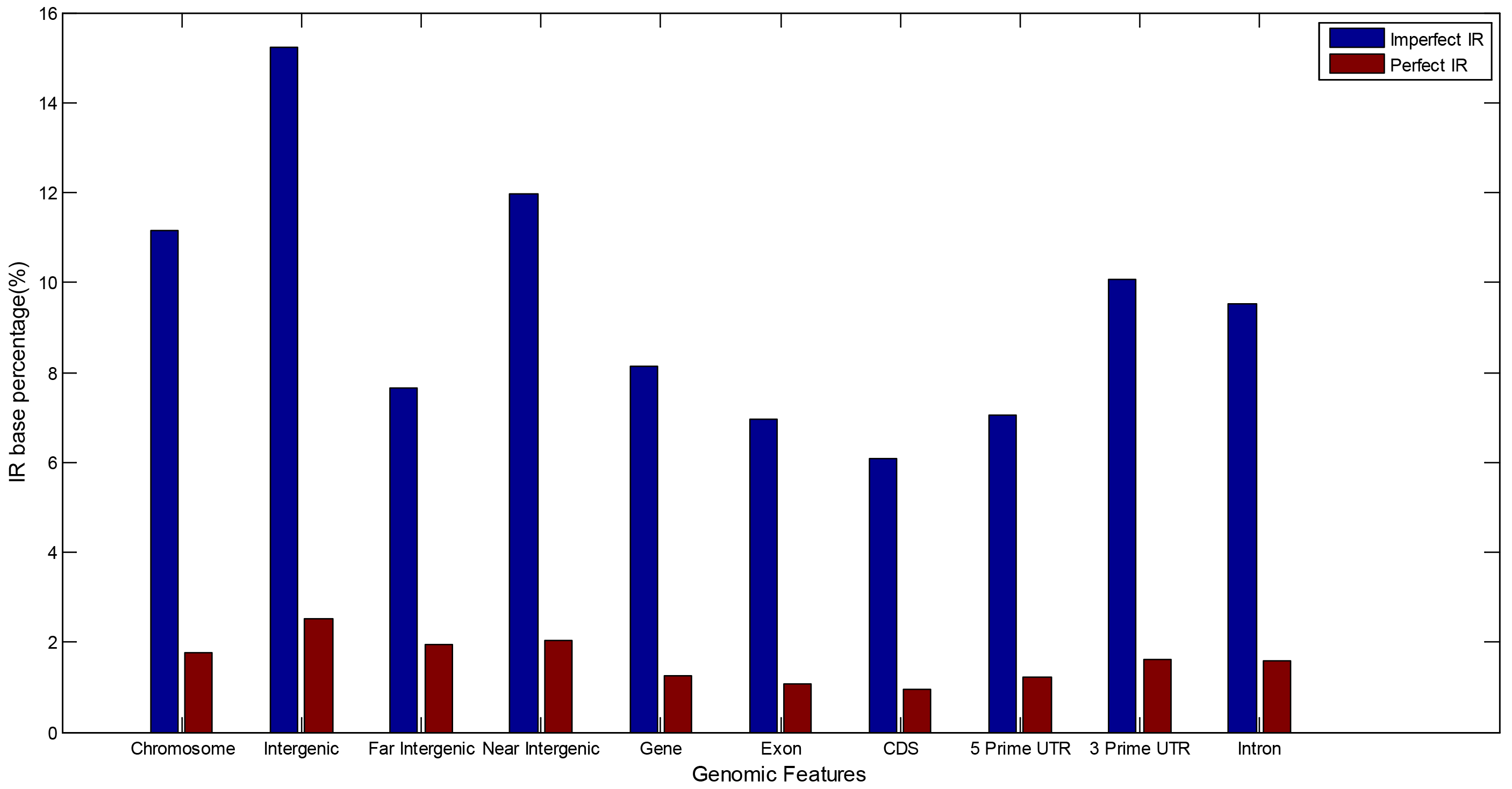


**Figure S1.11 The percentage of imperfect and perfect inverted repeat bases in various genomic regions across all chromosomes.**
